# The role of hospital midwives in the Netherlands

**DOI:** 10.1186/1471-2393-10-80

**Published:** 2010-12-09

**Authors:** Therese A Wiegers, Chantal WPM Hukkelhoven

**Affiliations:** 1 NIVEL (Netherlands institute for health services research), P.O.Box 1568, 3500 BN Utrecht, the Netherlands; 2 PRN (The Netherlands Perinatal Registry), P.O.Box 8588, 3503 RN Utrecht, the Netherlands

## Abstract

**Background:**

Most midwives in the Netherlands work in primary care where they are the lead professionals providing care to women with 'normal' or uncomplicated pregnancies, while some midwives work in hospitals ("clinical midwives"). The actual involvement of midwives in maternity care in hospitals is unknown, because in all statistics births in secondary care are registered as births assisted by gynaecologists. The aim of this study is to gain insight in the involvement of midwives with births in secondary care, under supervision of a gynaecologist. This is done using data from the PRN (The Netherlands Perinatal Registry), a voluntary registration of births in the Netherlands. The PRN covers 97% to 99% of all births taking place under responsibility of a gynaecologist.

**Methods:**

All births registered in secondary care in the period 1998-2007 (1,102,676, on average 61% of all births) were selected. We analyzed trends in socio-demographic, obstetric and organisational characteristics, associated with the involvement of midwives, using frequency tables and uni- and multivariate logistic regression analyses. As main outcome measure the percentage of births in secondary care with a midwife 'catching' the baby was used.

**Results:**

The proportion of births attended by a midwife in secondary care increased from 8.3% in 1998 to 26.06% in 2007, the largest increase involving spontaneous births of a second or later child, on weekdays during day shifts (8.00-20.00 hr) from younger mothers with a gestational age (almost) at term. After 2002, parallel to the growing numbers of midwives working in hospitals, the percentage of instrumental births decreased.

**Conclusions:**

In 2007 more midwives are assisting with more births in secondary care than in 1998. Hospital-based midwives are primarily involved with uncomplicated births of women with relatively low risk demographical and obstetrical characteristics. However, they are still only involved with half of the less complicated births, indicating that there may be room for more midwives in hospitals to care for women with relatively uncomplicated births. Whether an association exists between the growing involvement of midwives and the decreasing percentage of instrumental births needs further investigation.

## Background

Maternity care in the Netherlands is known for its high percentage of home births and for the independent position of midwives. In 2002 40.6% of all births took place in primary care, assisted by a midwife or general practitioner, 29.4% at home, 11.2% in hospital or birth centre, and 59.4% of all births took place in secondary care, under supervision of a gynaecologist in hospital [1].

The Dutch health care is organised in primary care, secondary care and tertiary care. For a patient the first point of contact with the health care system is primary care, which is freely accessible, close to people's homes, and general. Primary care providers as general practitioners (GPs) and midwives are gatekeepers for secondary care: hospitals and medical specialists. A patient needs a referral from a primary care practitioner to have access to a secondary care practitioner, a medical specialist who, in turn, can refer a patient to highly specialised tertiary care.

Most midwives in the Netherlands work in primary care where they are the lead professionals providing care to women with 'normal' or uncomplicated pregnancies. They are independent practitioners, like general practitioners or family doctors, and work in single-handed, duo-, or group-practices. In case of complications or an increased risk of complications during pregnancy, during labour or in the postpartum period, the midwife will refer her client to secondary care, where a gynaecologist will take over responsibility. The indications for referral have been agreed upon by all professional groups involved (gynaecologists, midwives and general practitioners) and are laid down in the so-called Obstetric Indication List [2]. A typical labour and maternity ward in a general hospital used to be staffed by obstetrical nurses, junior-doctors, sometimes a gynaecologist-in-training, and one or more gynaecologist/obstetricians, with occasionally a midwife.

In the last fifteen years the number of midwives practising in primary care has increased from 1.042 to 1.871 [3]. In addition, there have always been some midwives working in hospitals ("clinical midwives"), in academic settings and teaching hospitals, primarily to assist and coach gynaecologists-in-training. In the last fifteen years their number has increased threefold (from 192 to 573) with the largest increase since 2002 [3,4]. Several reasons are given for this increase. For instance, staff shortages on labour wards, leading to an increased demand for midwives to fill the vacancies of obstetric nurses and physicians-in-training. But also a growing preference among midwives for a salaried position with regular working hours (only possible in secondary care), especially after having been being self-employed in primary care for several years, during a period of increasing workload [5,6].

In the same period the attitude towards the role of midwives in secondary care has changed from assistant to semi-autonomous (but not independent) care provider. For instance, in 2000 the Dutch association of gynaecologists (NVOG) issued a statement, saying that the presence of midwives on the labour and delivery wards must: 'be seen in the light of an improvement of the quality of patient care on the delivery ward' [7]. And the Dutch association of midwives (KNOV) stated in 2002 that: 'the most important feature of the clinical midwife is that she, as specialist in physiological care, will guard the physiological approach of a patient with a medical indication within the clinical setting' [8]. More recently, in 2008, the NVOG stated that the clinical midwife is a valued addition to the obstetrical team because of her specific knowledge of the physiology of pregnancy, birth, and the puerperium [9].

Midwives working in hospitals have had the same 4-year vocational training at Bachelor level and have the same qualifications as primary care midwives in the Netherlands. That means they are qualified to assist healthy women with uncomplicated births only. But, because of working in a hospital as part of the obstetrical team and under supervision of a gynaecologist, they sometimes perform specific tasks or interventions - such as induction of labour - that are formally outside their competence. Since 2005 additional training, focussed on caring for women with pathology or an increased risk of complications, is available for midwives working in hospital. Since then, the debate about the role of hospital midwives has been between the value of their input as specialists in physiology - in which case they are expected to continue to be primarily involved in normal, uncomplicated labour and birth - and the value of enhancing their competency, so that some tasks of the gynaecologist can be shifted to the midwife, in which case they will increasingly be involved in more complicated births.

Until now, the actual involvement of midwives in maternity care in hospitals has remained invisible for outsiders, because in all statistics births in secondary care are registered as births assisted by gynaecologists. The aim of this study is to reveal the work of clinical midwives, to gain more insight in their role, and especially to determine whether that role is changing with the increase in the numbers of clinical midwives since 2002. We have three research questions. First: how many of the births in secondary care are assisted by midwives? Second: what are the characteristics of the women, the births, and the settings of midwife-assisted births in secondary care? And third: Are there any differences in the involvement of midwives before and after 2002?

## Methods

### Data source

The Netherlands Perinatal Registry (PRN) collects data from almost all births in the Netherlands. Perinatal data are collected in three separate registries: one for primary care (LVR1), one for secondary care (LVR2) and one for neonatal/paediatric care (LNR). In this retrospective study data of all births, registered in the LVR2 between 1 January 1998 and 31 December 2007, are used for analysis. The LVR2 registry starts at first contact (booking visit or referral from primary care) and contains complete perinatal data from 16.0 gestational weeks onwards. The coverage of the LVR2 is almost complete: in 1999 97% of all gynaecology partnerships provided data, in 2007 99%. Births registered in the LVR2 constitute 59% (1998) to 65% (2007) of all births occurring in the Netherlands [10]. In the Netherlands ethical approval is not required for this type of study (secondary analyses on anonymous data). Nevertheless the Steering Committee of the PRN approved this study. The PRN is registered at the Dutch Data Protection Authority.

### Outcome measurement and determinants

The outcome measure in the analyses was the type of caregiver 'catching' the baby, differentiated in 'gynaecologist', 'gynaecologist-in-training', 'midwife' and 'other' (GP, nurse, other). On the LVR2 registration form a distinction is made between the person responsible for the care provided at the time of birth and the person 'catching' the baby. With births occurring in secondary care the gynaecologist is always the responsible care provider, but not always present at the birth. The person 'catching' the baby is the one present at the time of birth and is assumed to be the one directly involved and thus the one assisting with the birth.

The socio-demographic, obstetric and organisational aspects used in the analyses included: maternal age, parity, cultural/ethnic background, gestational age, process of birth, time of birth, type of hospital, and moment of referral (before onset of labour or during labour).

### Statistical analysis

The number and frequency of births in secondary care, subdivided by different caregivers catching the baby, was presented for each year. We analysed which birth-related variables and background variables (maternal and hospital characteristics) were associated with the midwife being the person 'catching the baby'. This analysis was done for 1998 and 2007 separately to study the difference between both ends of the time scale. Following that, the data were combined in two periods: 1998-2002 and 2003-2007 to study the changes since 2002. Because the LVR2 contains nationwide population data and no sample data, differences between subgroups are absolute differences and statistical testing is not required.

For each socio-demographic, obstetric and organisational feature, the strength of its association with the outcome was first expressed as crude odds ratios (OR) with 95% confidence intervals (CI). We then adjusted the ORs in a multivariate logistic regression analysis to show the contribution of the examined feature in relation to the other characteristics. Interaction effects between parity and, subsequently, maternal age, process of birth and moment of referral were also examined with logistic regression analysis.

On average 9% of the records had missing data on the outcome measure (person catching the baby) and these records have been removed from the analyses. Zero to 2.3% of the characteristics were missing. Altogether, 3.2% of the babies from nulliparous mothers had missing values for one or more characteristics; for babies from multiparous mothers this percentage was 2.4%. These babies were not included in the multivariate logistic regression analysis.

All analyses were performed using a computer software package (SAS for Windows version 9.1; SAS Institute Inc., Cary, NC, USA).

### Details of ethics approval

Ethical approval is not required for this type of study in the Netherlands.

The PRN is registered at the Dutch Data Protection Authority.

## Results

Between 1998 and 2007 the proportion of births in secondary care increased from 59% to 65% of all births. Although the gynaecologist is the responsible care provider with all secondary care births, the person giving hands-on care is often someone else. In almost half of these births the gynaecologist-in-training was the one 'catching the baby', but since 2002 there is a strong increase in the number of births attended by a midwife. Table 1 shows an increase in the proportion of births attended by a midwife in secondary care from 8.3% in 1998 to 26.1% in 2007.

**Table 1:** Number of children born in secondary care (PRN data), by caregiver 'catching the baby'

year	nr of children born in secondary care (% of all children born)	care giver 'catching the baby': N (%)			
		
		gynaecologist	gyn-in-training	midwife	other (GP, nurse)
1998	112149 (0.59)	40120 (35.8)	49635 (44.3)	9304 (8.3)	13090 (11.7)
1999	109147 (0.57)	39421 (36.1)	49890 (45.7)	9106 (8.3)	10730 (9.8)
2000	116394 (0.60)	41308 (35.5)	56305 (48.4)	9814 (8.4)	8967 (7.7)
2001	113279 (0.61)	41994 (37.1)	55259 (48.8)	8016 (7.1)	8010 (7.1)
2002	108298 (0.60)	38272 (35.3)	55307 (51.1)	7901 (7.3)	6818 (6.3)
2003	111555 (0.62)	36842 (33.0)	54806 (49.1)	13699 (12.3)	6208 (5.6)
2004	108553 (0.62)	34208 (31.5)	52086 (48.0)	16594 (15.3)	5665 (5.2)
2005	105174 (0.63)	32213 (30.6)	49897 (47.4)	18668 (17.8)	4396 (4.2)
2006	108457 (0.64)	32215 (29.7)	48156 (44.4)	24186 (22.3)	3900 (3.6)
2007	109670 (0.65)	31178 (28.4)	46282 (42.2)	28577 (26.1)	3633 (3.3)

**total**	**1102676 (0.61)**	**367771 (33.4)**	**517623 (46.9)**	**145865 (13.2)**	**71417 (6.5)**

**Figure 1:**
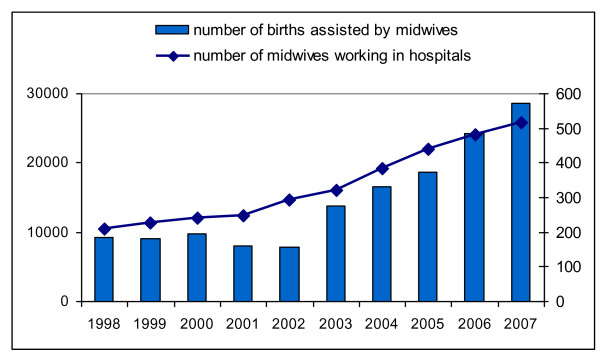
**Number of births in secondary care, assisted by midwives on the left-hand scale and the number of midwives working in hospitals on the right-hand scale**.

This increase in itself is no surprise, regarding the increasing numbers of hospital midwives. It merely illustrates that midwives are more and more replacing obstetrical nurses and GPs as well as gynaecologists as hands-on care givers on labour wards in secondary care. Figure 1 shows the relation between the number of midwives working in hospitals and the number of births in secondary care assisted by midwives. The correlation between these two is strong.

To get a clearer picture of the role of the midwife, a number of distinctions were made. First, we compared the outcome (midwife 'catching' the baby) in the group of women who were already in secondary care before the onset of labour and the group of women being referred during labour or birth. Both outcomes were comparable, with an increase of midwifery involvement from less than 10% in 1998 to approximately 25% in 2007, although there was a slightly (2-3%) larger involvement of midwives in the group of referrals during labour (Table 2).

**Table 2:** Birth related variables of births in secondary care assisted by a midwife (percentage of all births in secondary care)

		midwife 'catching the baby': (%)	
birth related variables		1998	2007
all births in secondary care		8.3	26.1
			
Referral	before onset of labour	7.3	24.5
	during labour	9.4	27.5
			
process of birth	spontaneous	13.3	41.0
	*- no induction, no augmentation*	*15.2*	*41.4*
	*- induction*	*11.3*	*41.5*
	*- augmentation*	*13.4*	*39.8*
	assisted (vacuum, forceps or CS)	0.2	1.9
			
time of birth	workday 8.00-20.00	8.6	26.9
	evening, night or weekend	8.0	25.3
			
time of spontaneous births	weekday 8.00-20.00	14.2	45.7
	evening, night or weekend	12.9	37.2

Second, as is also shown in Table 2 we compared the involvement of midwives with spontaneous births and with assisted births (vacuum, forceps or Caesarean Section (CS)). The midwives' involvement with spontaneous births has increased from 13% to 41%, while the proportion of spontaneous births of all births in secondary care varied only slightly, from 61% in 1998, via 56% in 2001 to 61% in 2007 (Table 3).

**Table 3:** Births in secondary care, by mode of intervention

year	children born in secondary care (% of all children born)	spontaneous, without induction or augmentation of labour	spontaneous with augmentation of labour	spontaneous with induction of labour	spontaneous (total)	assisted births (vacuum/forceps/CS)
	n*	**%**	**%**	**%**	**%**	**%**

1998	111547 (0.56)	0,30	0,11	0,19	0,61	0,39
1999	108941 (0.54)	0,30	0,12	0,19	0,60	0,40
2000	116208 (0.56)	0,29	0,12	0,18	0,59	0,41
2001	113022 (0.55)	0,27	0,11	0,18	0,56	0,44
2002	108153 (0.53)	0,28	0,12	0,17	0,56	0,44
2003	111470 (0.55)	0,29	0,12	0,16	0,58	0,42
2004	108412 (0.56)	0,29	0,13	0,15	0,58	0,42
2005	104930 (0.56)	0,29	0,14	0,15	0,59	0,41
2006	108137 (0.58)	0,30	0,15	0,15	0,60	0,40
2007	109285 (0.60)	0,29	0,17	0,16	0,61	0,39

**Total**	**1100105 (0.56)**	**0,29**	**0,13**	**0,17**	**0,59**	**0,41**

* differences with Table 1 are due to missing values on the intervention variable

**Figure 2:**
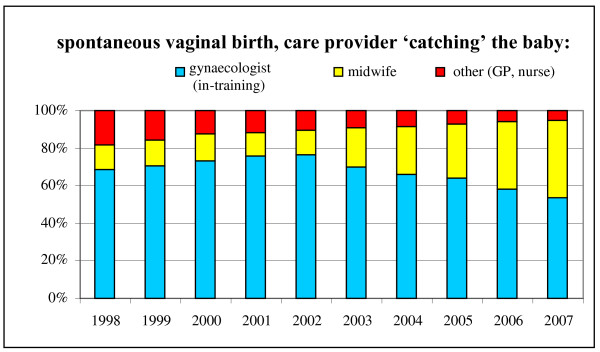
**Spontaneous vaginal births (on average 60% of all children born in secondary care), by care provider 'catching' the baby, in percentages**.

Figure 2 shows the increase of the percentage of births in which the midwife was the one catching the baby for spontaneous vaginal births only. Differentiating within all spontaneous births in secondary care between births with and births without induction and/or augmentation of labour showed a decrease in the rate of induction (from 32% in 1998 to 25% in 2007, not in table) while showing an increase in the involvement of the midwife with births with induction of labour (from 11% in 1998 to 42% in 2007) and births with augmentation of labour (from 13% in 1998 to 40% in 2007). This is in both cases approximately the same increase as with all spontaneous births.

Table 2 also shows that the distinction according to time of birth for all births does not make a difference: midwives are just as often involved during evenings, nights and weekends as they are during the daytime on weekdays. However, if both previous distinctions are combined, the data show that midwives are more often involved with spontaneous births in the daytime on weekdays, than at other times of the week.

Table 4 shows the increased involvement of midwives over time in relation to background variables of the women and the hospitals. Midwives were least involved with women of 40 years or older, with women giving birth for the first time, with very preterm births and with births in non-academic teaching hospitals. Cultural/ethnic background showed no difference. The differences between age groups, parity groups, gestational age groups and hospitals and the lack of difference in the cultural/ethnic background groups, seen in 1998, were repeated in 2007.

**Table 4:** Characteristics of women giving birth in secondary care assisted by a midwife and of the hospitals

		midwife 'catching the baby': (%)	
background variables		1998	2007
all births in secondary care		8.3	26.1
			
Age	< 20 yr	10.1	31.7
	20-40 yr	8.3	26.1
	≥ 40 yr	6.6	22.5
			
Parity	0	6.5	21.8
	1	9.7	27.5
	2+	10.1	30.5
			
cultural/ethnic background	western	8.2	26.1
	non-western	8.9	26.2
			
gestational age	37-< 42	8.9	27.2
	32-< 37	6.7	23.2
	< 32	3.0	10.4
			
Hospital	academic hospital	9.3	24.7
	teaching hospital (not academic)	2.2	17.1
	non-teaching non-academic	11.3	36.1

To analyse the associations between socio-demographic, obstetric and organisational characteristics and outcome, the data were combined in two periods: 1998-2002 and 2003-2007. After controlling for background variables, midwives were more likely to assist with spontaneous births without induction or augmentation occurring in the daytime during weekdays and in academic and non-teaching hospitals (Table 5). We observed interaction between parity and gestational age, process of birth and referral respectively. Therefore the logistic regression analysis was performed for nulliparous and multiparous women separately.

**Table 5:** Associations between socio-demographic, obstetric and organisational characteristics and outcome (midwife catching the baby)

	Nulliparae		Multiparae	
	1998-2002	2003-2007	1998-2002	2003-2007
Characteristics	OR* multivariable		OR* multivariable	
	(95% CI^#^)		(95% CI^#^)	
referral				
- before the onset of labour (reference)	1.00	1.00	1.00	1.00
- during labour	1.23 (1.18-1.28)	1.02 (0.99-1.05)	1.54 (1.48-1.60)	1.12 (1.08-1.16)
process of birth				
- spontaneous, no induction, no augmentation (reference)	1.00	1.00	1.00	1.00
- induction	0.74 (0.70-0.77)	0.78 (0.75-0.81)	0.78 (0.75-0.81)	0.83 (0.80-0.86)
- augmentation	0.81 (0.77-0.84)	0.80 (0.77-0.82)	0.79 (0.76-0.83)	0.79 (0.76-0.82)
- vacuum/forceps/CS	0.01 (0.01-0.02)	0.03 (0.03-0.03)	0.01 (0.01-0.01)	0.01 (0.01-0.01)
time of birth				
- weekday 8.00-20.00 (reference)	1.00	1.00	1.00	1.00
- evening, night of weekend	0.83 (0.80-0.86)	0.71 (0.70-0.73)	0.82 (0.80-0.85)	0.75 (0.73-0.77)
Maternal age				
- < 25	1.03 (0.98-1.07)	1.05 (1.02-1.08)	0.97 (0.91-1.03)	1.02 (0.97-1.08)
- 25-< 35 (reference)	1.00	1.00	1.00	1.00
- ≥ 35	0.96 (0.91-1.03)	0.93 (0.89-0.97)	0.99 (0.95-1.02)	0.95 (0.92-0.98)
Ethnicity				
- western (reference)	1.00	1.00	1.00	1.00
- non-Western	1.16 (1.10-1.22)	1.07 (1.03-1.11)	1.23 (1.19-1.28)	1.04 (1.01-1.08)
Gestational age				
- < 32 weeks	0.24 (0.21-0.28)	0.20 (0.18-0.22)	0.24 (0.21-0.28)	0.24 (0.21-0.27)
- 32-< 37 weeks	0.59 (0.55-0.62)	0.63 (0.61-0.66)	0.57 (0.54-0.61)	0.61 (0.58-0.64)
- 37-< 42 weeks (reference)	1.00	1.00	1.00	1.00
- ≥ 42 weeks	1.12 (1.04-1.19)	1.19 (1.13-1.25)	1.12 (1.06-1.19)	1.21 (1.15-1.27)
Type of hospital				
- academic	8.62 (8.01-9.28)	2,12 (2,02-2,21)	8,80 (8.46-9.52)	3.29 (3.16-3.43)
- teaching (reference)	1.00	1.00	1.00	1.00
- non-teaching	8.21 (8.02-9.28)	3.50 (3.41-3.60)	7.55 (7.18-7.94)	4.09 (3.97-4.21)

* OR = Odds Ratio, via multivariable logistic regression analysis (adjusted for the characteristics mentioned above and year of birth).

**^#^**95% CI = 95% Confidence Interval. If this interval does not include the value 1, the factor has a statistically significant effect on outcome

The analysis showed that nulliparous women were more often assisted by midwives, when referred during labour, in the earlier period (1998-2002), but not in the later period (2003-2007). In the case of multiparae there is no difference between both periods, midwives were more often assisting women after referral during labour than after referral before the onset of labour. In both periods and for both groups of women midwives were less often assisting non-spontaneous births and births during out-of-office hours. The differences regarding different age groups and ethnicity are small. In both groups of women and in both time periods midwives are less often involved in pre-term birth and more often in post-term birth. In the later period the involvement of midwives in the different types of hospitals diverged less from each other than in the earlier period. That may be due to the fact that only after 2002 a considerable number of midwives were employed by non-teaching hospitals.

**Figure 3:**
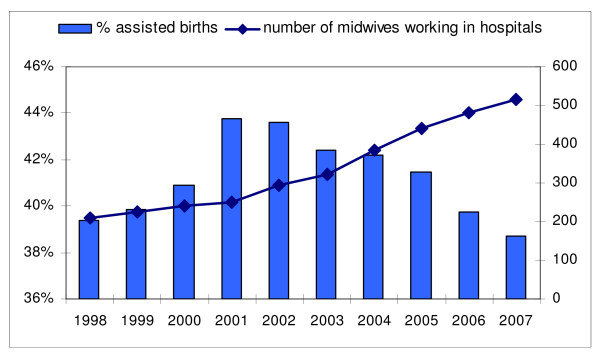
**Percentages of assisted births (vacuum/forceps/CS) in secondary care on the left-side scale and the number of midwives working in hospitals on the right-side scale**.

To find out whether the involvement of midwives with births in secondary care can help to prevent interventions, a prospective study is needed. But we did find that the steady increase after 2001 of the number of midwives working in hospitals coincided with a decrease in the percentage of instrumental births in secondary care (Table 3). This is not simply the result of an increasing number of births in secondary care, with a stable number of interventions, or of an increasing proportion of births in secondary care, with a stable proportion of assisted births, calculated over all births. Because referral to secondary care is regarded as an indication that an intervention is needed, the proportion of assisted births within secondary care, in stead of the proportion of assisted births calculated over all births, is expected to stay more or less equal or to increase, but certainly not to decrease. As can be seen in Table 1 and 3, the number of births in secondary care first increased, with a peak of 116,000 children born in 2000, then decreased again. The number of assisted births (only occurring in secondary care) followed a similar pattern, with an increase leading to a peak in 2001, followed by a steady decrease as shown in Table 3 and the proportion of births in secondary care steadily increased. However, the rate of assisted births changed, not only in secondary care as is shown in Figure 3 but also in the population at large. The percentage of instrumental births, calculated on all births in the Netherlands decreased from 26,6% in 2001, via 25,6% in 2004, to 24,9% in 2007 (results not shown).

## Discussion

This study has shown not only that more midwives were assisting with more births in secondary care in 2007 than they were in 1998, but also that their involvement was primarily with spontaneous births and births during day shifts rather than with complicated births and births during night or weekend shifts. We also found that increased involvement of midwives coincides with a decrease in the percentage of instrumental births in secondary care. To appreciate this it is important to realise that all women giving birth in secondary care have been referred to a gynaecologist because of perceived risks or complications. And this status of no longer being considered as low-risk, may trigger an interventionist response. In many cases interventions are indeed needed, but yet some, and in this dataset an increasing number, experience an uncomplicated birth, because the perceived risk did not manifest itself or could successfully be prevented.

We now can answer the research questions we formulated. The first was: how many of the births in secondary care are assisted by midwives? We found that the number has increased from 8 percent to 26 percent of all births in secondary care.

The second research question was: what are the characteristics of the women, the births, and the settings of midwife-assisted births in secondary care? The profile of a midwife-assisted birth in secondary care is that of a spontaneous birth, taking place on a weekday during office hours, in a non-teaching hospital. This profile reflects not only the formal competence of a midwife, but possibly also their work arrangements: primarily working during office hours and much less during evenings, nights and weekends. This profile also suggests that midwives have kept to their formal competence and have not been extending that by increasingly being involved with more complicated births.

The third question we posed was: Are there any differences in the involvement of midwives before and after 2002? The difference we found was in the quantity, not in the characteristics of the women or the births: there was no real difference between the groups of women served by midwives in the two time periods. Hospital midwives were before and after 2002 primarily involved with uncomplicated, spontaneous births. However, concurrent with the increasing involvement of midwives we found a decreasing number of instrumental births. Whether these two developments are related still has to be proven. There are indications from other studies that a relation exists. For instance, in a recent Cochrane review, comparing midwife-led care with other models of care (medical-led care or shared care), midwife-led care was associated with several benefits for mothers and babies, such as fewer episiotomies or instrumental births and increased chance of having a spontaneous vaginal birth and initiating breastfeeding [11,12]. Although the midwives working in hospitals in the Netherlands are not working in midwife-led care, their presence in the clinical setting may introduce elements of the midwifery model, such as increased continuity of care and reduction of unnecessary technology.

Our results show that the involvement of hospital midwives with spontaneous vaginal births has increased from 13% to 41%. That means however, that they are still not involved with more than half of the less complicated births, being approximately 60% of all births in secondary care, so there may be plenty of room for more midwives in hospitals.

### Limitations of this study

This is a retrospective study, using existing data, collected in a standard form, not designed for these specific research questions. First, the variable used to indicate the involvement of a caregiver is the person 'catching' the baby. There is no absolute certainty that this person is indeed the one most closely involved with the birth, but it is the only variable available to differentiate between the caregiver responsible for the birth (i.e. the gynaecologist) and the person attending the woman. For instance, the assisting midwife or gynaecologist (in-training) may have let the partner catch the baby, which would have been noted as 'other'. On the other hand, some women giving birth in secondary care after referral during labour may have been attended by their own, primary care midwife. These incidences might be confounding the analysis somewhat, because we do not know how often that may have happened.

Second, there is no detailed information available about the hospital midwives in the registration used for this project. For example, we do not know how many of them work only in daytime shifts or in 24-hour shifts.

## Conclusion

The analyses have shown that, although the involvement of midwives with births in secondary care has increased, they are primarily involved with relatively uncomplicated births and not with the more complicated births. But there are still twice as much uncomplicated births in secondary care not assisted by midwives. This may indicate that there is plenty of room for more midwives in hospitals. The analyses have also shown that, since 2002 the percentage of assisted births (vacuum, forceps, CS) has decreased, not only in secondary care but also when calculated as a proportion of all births registered in the Netherlands Perinatal Registry. The interesting question is, whether these two developments are related, but that still needs to be analysed. That analysis is not possible with these retrospective data.

These analyses are only a first attempt to shed more light onto the role of midwives working in hospitals in the Netherlands. For future studies more information is needed about the midwives in the different hospitals, their education, their attitudes and their actual involvement with births in secondary care.

## Competing interests

The authors declare that they have no competing interests.

## Authors' contributions

Both authors contributed substantially to the design of the study. C.W.P.M.H. analysed the data, T.A.W. prepared the manuscript. Both authors read and approved the final manuscript.

## Pre-publication history

The pre-publication history for this paper can be accessed here:

http://www.biomedcentral.com/1471-2393/10/80/prepub
